# Sleep problems and referral intentions in mental health services: service user self-report and staff proxy report surveys

**DOI:** 10.1186/s12888-023-04817-6

**Published:** 2023-08-10

**Authors:** Sophie M. Faulkner, Richard J. Drake, Emily Eisner, Penny E. Bee

**Affiliations:** 1 Faculty of Biology, Medicine and Health, The University of Manchester, Oxford Rd, Manchester, M13 9PL UK; 2 Greater Manchester Mental Health NHS Foundation Trust, Bury New Rd, Prestwich, Manchester, M25 3BL UK

**Keywords:** Sleep, Circadian rhythm, Insomnia, Obstructive sleep apnoea, Mental health services, Psychosis, Behavioural therapies, CBTi, Light exposure, Implementation theory, Survey

## Abstract

**Background:**

Sleep problems are common in mental health service users, but few non-pharmacological therapies are offered. Therapies are being developed and tested, but there may be barriers to these therapies reaching those who need them.

**Methods:**

Light-Dark and Activity Rhythm Therapy (L-DART), is a new sleep therapy delivered by an occupational therapist, which has been feasibility tested in people with schizophrenia spectrum diagnoses. This paper presents two surveys, conducted with mental health staff and service users, on sleep problems, treatment wishes; and barriers and facilitators to uptake of L-DART or similar therapies. Descriptive statistics, single-level and multi-level ordinal logistic regression were used to examine factors associated with sleep problems and referral intentions.

**Findings:**

Sleep problems were commonly identified by staff and service users, there was demand for non-pharmacological intervention across diagnostic and demographic categories, but staff readiness to refer differed according to NHS Trust and service user diagnosis. Staff and service user reports differed in awareness of sleep disordered breathing and parasomnias, and wish for referral. Staff were more confident identifying sleep problems than addressing them, but more training was associated with greater confidence concerning both assessment and treatment.

**Conclusions:**

A range of sleep problems are prevalent and recognised in mental health service users, and there is an unmet need for non-pharmacological sleep interventions. Improving suitable resources to support self-management in this group may help; Staff and service users also reported a high readiness to refer or be referred for sleep interventions. Staff training to improve identification of sleep problems, and differentiation between types of sleep problems, would support access to the most appropriate treatments.

**Supplementary Information:**

The online version contains supplementary material available at 10.1186/s12888-023-04817-6.

## Highlights

Self-reported or staff reported sleep problems are common in mental health service users.

Staff are less aware of sleep disordered breathing and parasomnias than are service users.

There is demand for non-pharmacological sleep interventions and wish to refer/be referred.

Staff have had limited training regarding sleep, but more training is associated with greater confidence to assess and address sleep.

**Supplementary Information:**

The online version contains supplementary material available at 10.1186/s12888-023-04817-6.

## Background

Sleep problems are common in people who use specialist mental health services [1], but mental health professionals express limited knowledge and confidence to address them [2–4]. The difficulty of addressing sleep problems in this group is increased as the types of sleep problems experienced are more diverse [5]. Insomnia is common, but people with bipolar or schizophrenia also frequently experience circadian dysregulation and excessive sleepiness [6–8]. Rates of obstructive sleep apnoea (OSA) and restless leg syndrome are also much higher in people with severe mental illness than in the general population [9, 10].

Research suggests improving sleep in people experiencing psychosis could reduce symptoms and improve functioning [8], but individuals may not always seek and/or access sleep treatment. Service-users sometimes accept sleep problems as an inevitable part of their condition [11, 12], don’t see sleep as a concern of mental health professionals [12], or feel ‘talking’ would have limited effect on their sleep [13].

Occupational therapists are an appropriate staff group to address sleep problems in mental health service users, utilising their relevant core skills [14–16], although this has not traditionally been a focus for the profession [16]. The authors have developed an occupational therapy intervention to improve sleep in people with schizophrenia spectrum diagnoses, called Light-Dark and Activity Rhythm Therapy (L-DART). L-DART differs from traditional cognitive behavioural therapy for insomnia in its increased emphasis on light exposure modification, and its personalisable pathways to treat not just insomnia but also hypersomnia, circadian rhythm disorders, or mixed presentations. Although developed with a focus on people with schizophrenia spectrum diagnoses, participants in the intervention development study suggested L-DART may be relevant trans-diagnostically [17], thus this study has focused on service users of mental health services more broadly. Feasibility testing has been completed and will be published separately [18].The MRC framework for complex interventions recommends advance consideration and investigation of issues which may affect larger scale testing, and suggests planning for potential barriers to implementation [19, 20]. Implementation theories suggest identifying what may prevent, or would facilitate, the desired behaviour from stakeholders [21, 22]. Critical factors which can affect intervention uptake include the intervention source, and the perceived relative importance of the targeted problem [23]. Some clinicians and academics suggested that poor understanding of the role of occupational therapists [24–26] could cause reluctance to refer, and some suggested service users and staff might find it difficult to identify sleep issues or seek help for them.

We present two cross-sectional surveys of staff and service users, evaluating presence of and awareness of different types of sleep problems, readiness to refer or be referred, and staff and service user factors influencing these.

## Methods

### Aims

The study aimed to:

1. examine staff confidence and knowledge to identify and address sleep problems.
2. examine the presence of and awareness of different types of sleep problems.
3. examine readiness to refer or be referred to an intervention like L-DART.
4. explore factors influencing help-seeking, and service user treatment preferences.

A favourable ethical opinion was obtained from Research Ethics Committee Reference 20/NW/0059.

### Participants

Staff and service users were recruited from two large mental health NHS Trusts in the North of England. Participation was anonymous.

### Inclusion, exclusion and recruitment

All clinical staff were eligible, and there were no exclusions. Quotas were used to attempt to balance staff groups included [27], and purposive sampling targeted remaining gaps later in recruitment by appealing for required staff groups. Posters, internal emails, social media, and presentations at meetings or trainings were used.

Any service user from a participating NHS Trusts could participate. There were no further inclusion or exclusions criteria, except not inviting anyone who involved staff felt would find completing the survey confusing or distressing. Recruitment was via meetings, leaflets/posters, phonecall/text, and in person from specific services (e.g. depot clinic, clozapine clinic, residential and inpatient settings).

### Survey development

Surveys questions covered factors that might influence service users’ sleep problems or readiness to be referred, such as age, gender, diagnosis, and sleep-related beliefs endorsed in prior research [12, 28, 29]. Although we considered embedding psychometrically tested self-report sleep assessments within the service user survey, we prioritised brevity, and the ability to present the same questions (with same phrasing) to staff and service users.

The staff and service user surveys were piloted, commented on and timed, with input from four clinical colleagues, and three patient public contributors respectively. Input was received from two ongoing patient public contributors and one person as a one-off. Input influenced removal of an item attempting to describe service receipt which was difficult to complete, and other more minor changes to phrasing.

### Survey structure and questions

The surveys are available in Appendix A. Service users were asked about their sleep problems (if any), their self-identified treatment needs, readiness and preferences, and demographic questions. We phrased sleep disorder questions in such a way as to obtain affirmative answers only from participants who recognised in themself either a sleep symptom or a sleep disorder, and did not ask about risk factors in order to assess prevalence, as this has been studied elsewhere using appropriate methods.

Staff were asked their profession, service, duration of experience, and knowledge and confidence to identify and address sleep problems. Staff were then asked to give a proxy report of sleep problems (if any) using identical terminology as used with service users, and readiness to refer, for the last 5 service users on their caseload with whom they had had contact. No personally identifying information was collected.

### Data collection

Both surveys were online, but service users were offered alternatives (paper, in person, or over the phone) to minimise sampling bias [30].

### Data analysis

Statistical analyses were conducted in Stata (version 14.0) and considered statistically significant at two-tailed p < 0.05. Multiple-choice responses were examined using descriptive statistics. Categories containing few participants were collapsed for analysis where appropriate. Severity, frequency and impact of sleep disruption were highly correlated with each other, and were consolidated into ordinal ‘sleep problem’ variables, consisting of the sum of severity, frequency and impact, for use in all subsequent analyses.

Ordinal logistic regression was used to examine which variables from the service user survey predicted more severe self-reported sleep problems, and which variables predicted desire for referral to a behavioural sleep intervention [31]. Multilevel ordinal logistic regression was used to examine the equivalent research questions in the staff survey, to account for the clustered nature of this data (as each staff participant reported on five service users) [32]. Free-text questions were analysed using a framework approach [33], coded by one researcher, in Nvivo software (version 12).

## Results

### Sample

We recruited 147 staff and 190 service users, meeting targets for all staff sub-groups except psychiatry trainee doctors (target = 3, recruited = 0). Staff participants reported on 619 anonymous service users. See Table 1 for demographics of both samples.

To evaluate indications of bias the demographics of service user survey participants were compared to service users of Trust #1 using summary information available from Business Intelligence. Both trusts have somewhat similar demographics. Ethnicity, gender and age were comparable, except for underrepresentation of people aged 71 years and over. See Appendix B.

**Table 1:** Demographics of service user and staff participants, and service users proxy reported on by staff

Service user participants	Total: 190	Staff participants	Total: 147	Service users via staff proxy	Total: 619
Primary diagnosis:		Profession		Primary diagnosis	
schizophrenia	83 (43.7%)	NHS support staff (clinical)	21 (14.4%)	schizophrenia spectrum disorder	199 (32.2%)
schizoaffective disorder	10 (5.3%)	clinical psychologist	12 (8.2%)		
bipolar affective disorder	14 (7.4%)	clinical psychology trainee	1 (0.7%)	bipolar affective disorder	53 (8.6%)
depression / anxiety	22 (11.6%)	mental health nurse	55 (38.7%)	depression / anxiety	102 (16.5%)
other psychosis diagnosis	16 (8.4%)	occupational therapist	12 (8.2%)	psychosis not otherwise specified	74 (12.0%)
personality disorder	13 (6.8%)	other AHP**	3 (2.1%)	personality disorder	77 (12.4%)
other, none of the above	7 (3.7%)	other junior doctors***	3 (2.1%)	substance misuse disorder	56 (9.1%)
I don’t have a diagnosis	10 (5.3%)	psychiatry consultant	15 (10.3%)	none of the above	58 (9.4%)
I don’t know / can’t remember	6 (3.2%)	psychiatry / other trainee	0 (0.0%)	**How often seen?**	
no response	9 (4.7%)	social worker	21 (14.4%)	more than weekly	146 (23.6%)
Ethnicity:*		student (other)	3 (8.3%)	weekly	89 (14.4%)
White British	140 (77.3%)	third sector (non-NHS)	0 (0.0%)	fortnightly	124 (20.0%)
Mixed	13 (7.2%)	**Experience**: mean, (SD)		3 weekly	53 (8.6%)
Asian	10 (5.5%)	Years in current profession	11.30 (10.2)	monthly	97 (15.7%)
Black/African/Caribbean	5 (2.8%)	Years in mental health	13.17 (10.0)	2–6 monthly	62 (10.0%)
Other white	4 (2.2%)	**Service**:	n= (%)	7 monthly - yearly	21 (3.4%)
Any other ethnic group	3 (1.7%)	CMHT****	66 (44.9%)	**How long known?**	
I prefer not to say	6 (3.3%)	Early Intervention in Psychosis	16 (10.9%)	less than 1 month	90 (14.5%)
**Gender**:		Rehabilitation	9 (6.1%)	1–3 months	120 (19.4%)
female	89 (48.9%)	Acute Inpatients	28 (19.0%)	> 3 months < 6 months	97 (15.7%)
male	86 (47.2%)	Crisis Team	2 (1.4%)	6 months − 1 year	116 (18.7%)
other*	5 (2.8%)	Other specialist service	20 (13.6%)	over 1 year	176 (28.4%)
I prefer not to say	2 (1.1%)	None of the above	6 (4.1%)		
**Age**		**Organisation**: n= (%)			
18–24	11 (6.0%)	NHS Trust #1	82 (55.8%)		
25–30	19 (10.4%)	NHS Trust #2	63 (42.9%)		
31–40	39 (21.4%)	Neither of the above	1 (0.7%)		
41–50	52 (28.6%)	Prefer not to say	1 (0.7%)		
51–60	42 (23.1%)				
61–70	16 (8.8%)				
71+	3 (1.7%)				

* non-binary / gender fluid / genderqueer / other / I prefer to self-describe ** (includes speech and language, dietician, physiotherapist, art/music/drama therapists) *** (staff grade, locum etc.) **** Community Mental Health Team

### Prevalence and severity of sleep problems

**Fig. 1:**
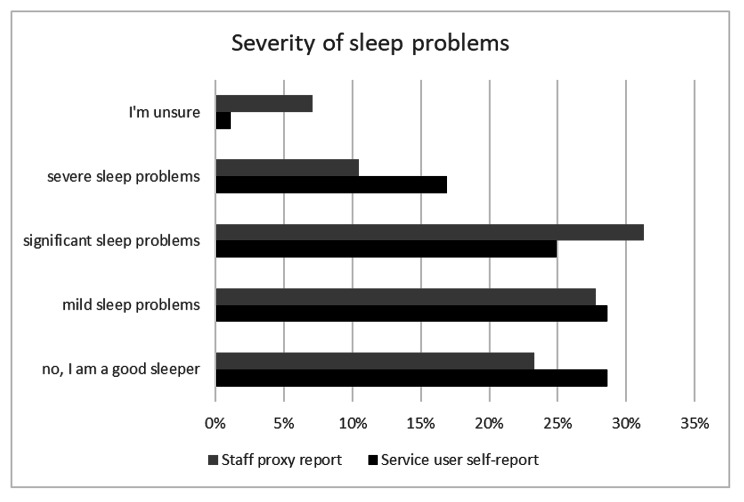
Severity of sleep problems reported

Most service users reported sleep problems (70.4%), and staff proxy-report was similar (69.6%). A large proportion reported significant or severe problems (service users 41.8%, staff proxy-report 43.39%). Service users endorsed ‘severe’ sleep problems slightly more often than staff proxy. See Fig. 1; Table 2.

Ordinal logistic regression of service user self-reports in a model including only diagnostic predictor variables suggested schizophrenia was associated with less severe sleep problems (4.8% severe, OR 0.383, p = 0.003). Personality disorder (38.5% severe, odds ratio (OR) 5.432, p = 0.001) or bipolar (35.7% severe, OR 3.475, p = 0.007) diagnoses were associated with worse sleep.

Goodness of fit statistics indicated that a model in which age, gender and NHS Trust were included as covariates provided a better fit then diagnostic variables alone, and explained more variance in sleep problems (with age gender and Trust: AIC 693, BIC 771, McFadden’s pseudo-R^2^ 0.119) (diagnostic variables only: AIC 773, BIC 839, pseudo-R^2^ 0.095). Once age, gender, and NHS Trust were added to the model, only diagnoses of bipolar (OR 2.741, p = 0.033) and personality disorder (OR 4.970, p = 0.004) remained statistically significant, schizophrenia did not predict better sleep; instead being from NHS Trust #2 predicted better sleep (OR 0.352, p = 0.003). Of note, proportionately more service user participants with schizophrenia were recruited from NHS Trust #2 (66.7% vs. 22.7%%, see Appendix C) and in NHS Trust #2 more were recruited from clozapine clinics.

Staff reported only 25.63% of their service users with schizophrenia spectrum disorders to be good sleepers, compared to > 40% in self-report responses above. Multilevel ordinal logistic regression of staff proxy reports found no significant associations between service user diagnosis and sleep problems, but certain professional groups were more likely to report sleep problems in their service users (clinical psychologists OR 3.239, p = 0.007, consultant psychiatrists OR 2.842, p = 0.009, occupational therapists OR 2.092, p = 0.045, and other allied health professionals OR 8.715, p = 0.034).

### Types of sleep problems

**Fig. 2:**
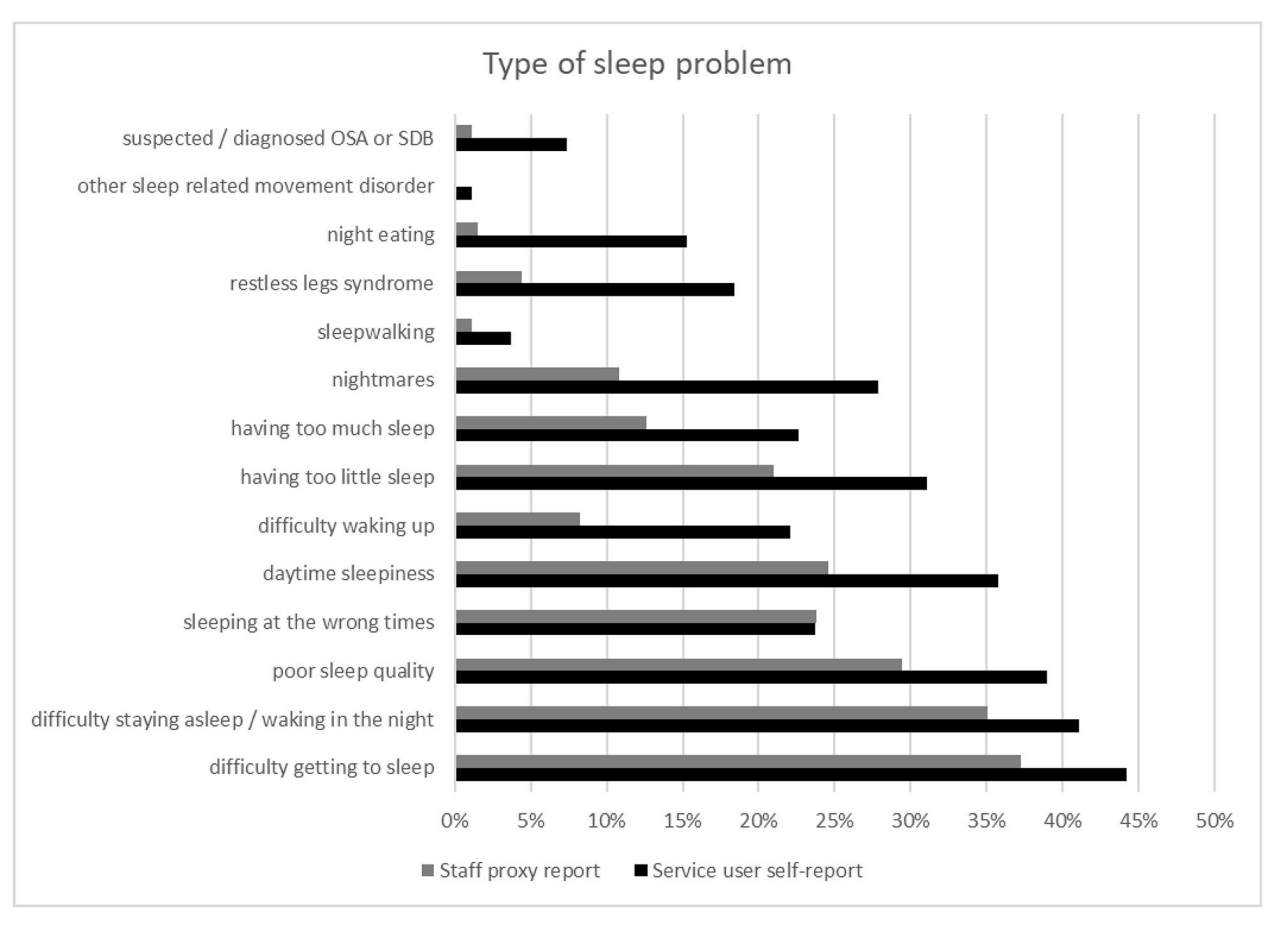
Types of sleep problems reported

The most common type of sleep problems reported by either group were ‘difficulty getting to sleep’ and ‘difficulty staying asleep / waking in the night’, with service users and staff proxy reporting similar levels of these. Service users reported significantly more daytime sleepiness, difficulty waking up, or having too much sleep, than staff proxy. Service users also reported significantly more night-eating, sleepwalking, restless legs syndrome, and OSA than staff proxy. See Fig. 2; Table 2.

**Table 2:** Differences between service user and staff proxy reports

Severity of sleep problems	Difference in proportions (service users – staff proxy)	95% CI of difference in proportions	z test of difference of 2 proportions		
			z=	p=	
no, I am a good sleeper	0.05	-0.02, 0.13	1.350	0.177	
mild sleep problems	0.01	-0.07, 0.08	0.078	0.94	
significant sleep problems	-0.06	-0.14, 0.01	-1.650	0.099	
severe sleep problems	0.06	0.01, 0.12	2.247	0.025	
I’m unsure	-0.06	-0.09, -0.03	-3.153	0.002	
**Type of sleep problem (select all that apply)**					
difficulty getting to sleep	0.07	-0.01, 0.15	1.604		0.109
difficulty staying asleep / waking in the night	0.06	-0.02, 0.14	1.401		0.161
poor sleep quality	0.10	0.02, 0.17	2.372		0.018
sleeping at the wrong times	0.00	-0.07, 0.07	0.000		1.000
daytime sleepiness	0.11	0.04, 0.19	2.938		0.003
difficulty waking up	0.14	0.08, 0.20	5.103		< 0.001
having too little sleep	0.10	0.03, 0.17	2.755		< 0.006
having too much sleep	0.10	0.04, 0.16	3.266		0.001
nightmares	0.17	0.10, 0.24	5.664		< 0.001
sleepwalking	0.03	0.00, 0.05	2.039		0.041
restless legs syndrome	0.14	0.08, 0.20	6.209		< 0.001
night eating	0.14	0.09, 0.19	7.666		< 0.001
other sleep related movement disorder	0.01	cannot calculate	cannot calculate		
suspected / diagnosed obstructive sleep apnoea / sleep disordered breathing	0.06	0.02, 0.10	4.464		< 0.001

### Staff training, confidence & knowledge

Levels of self-reported staff sleep training were low, and knowledge from informal sources was more common than formal training. Most staff were relatively confident in their ability to identify sleep problems, but less confident in intervening (see Table 3). Staff were understandably more often ‘unsure’ of service user’s sleep problems, than service users were themselves (see Table 2), but this included being unsure when the service user was seen often or known for years (see Appendix C).

Greater staff confidence in identifying sleep problems was significantly associated with having had more sleep training (OR 2.011, p = 0.006). Lower confidence identifying sleep problems was associated with working in an ‘other specialist service’ (OR 0.198, p = 0.027), or being a psychiatry consultant doctor (OR 0.131, p = 0.017). Greater confidence to address or improve sleep problems was also significantly associated with having more sleep training (OR 2.093, p = 0.002), whereas lower confidence was associated with being a psychiatry consultant doctor (OR 0.196, p = 0.033), working in an acute inpatient setting (OR 0.306, p = 0.025), and working in rehabilitation (OR 0.110, p = 0.016).

**Table 3:** Staff confidence and knowledge regarding identification and treatment of sleep problems

Statement	not confident at all	not very confident	reasonably confident	very confident
How confident do you feel in your ability to identify sleep problems?	0 (0%)	16 (10.9%)	102 (69.4%)	29 (19.7%)
How confident do you feel in your ability to address or improve sleep problems?	4 (2.7%)	56 (38.1%)	77 (52.4%)	10 (6.8%)
	**none**	**hardly any**	**some**	**a lot**
How much training have you had regarding sleep?	47 (32.0%)	48 (32.7%)	49 (33.3%)	2 (1.4%)
**Please select the main sources of your knowledge (select all that apply)**				
I have a sleep related qualification		0 (0%)		
my own research		108 (73.5%)		
general awareness and mainstream media		73 (49.7%)		
content covered as part of my professional training (pre-qualification)		55 (37.4%)		
a university / college course about sleep		7 (5.0%)		
Trust delivered training about sleep		19 (12.9%)		
a course from another provider		11 (7.5%)		

### Seeking treatment

Both surveys briefly described the L-DART intervention (“a non-drug sleep intervention involving changes to sleep schedule, light exposure and daytime occupation/activity […] from a mental health occupational therapist”) and asked participants hypothetically, whether they would refer or be referred to such an intervention. For service users with any sleep problem (mild, significant, or severe), most staff said they would ‘definitely’ or ‘probably’ refer (62.9%), and 46.5% of service users said they would want to be referred. See Table 4.

**Table 4:** Staff and service user referral intentions (if mild, significant or severe sleep problems), and service user treatment beliefs and preferences

Would you refer / want to be referred?	Service user referral intent	Staff referral intent		z test of difference of 2 proportions		
				z=		p=
total asked (asked sleep problems mild- severe)	134	429				
definitely	45 (33.8%)	136 (31.7%)		-0.301		0.763
probably	24 (12.6%)	134 (31.2%)		2.638		0.004
maybe	23 (17.3%)	61 (14.2%)		-0.696		0.486
probably not	14 (10.5%)	57 (13.3%)		0.715		0.475
no / highly unlikely	27 (20.3%)	41 (9.6%)		-3.133		0.002
**Are any of these statements true for you? (service-user responses)**				(select all that apply) n (%)		
I don’t want to improve my sleep				42 (22.1%)		
I don’t think it’s possible to improve my sleep				47 (24.7%)		
I would like to improve my sleep on my own				41 (21.6%)		
I would like self-help advice				37 (19.5%)		
I would like help from a professional to improve my sleep				61 (32.1%)		
I would like a therapy to help improve my sleep				53 (27.9%)		
**Statement (service user responses)**	**Strongly agree**	**Agree**	**Neither agree nor disagree**	**Disagree**	**Strongly disagree**	
I would like to be prescribed a drug for my sleep	15.6%	18.0%	18.8%	17.2%	30.5%	
I am already taking a drug for my sleep	18.1%	16.5%	9.5%	14.2%	41.7%	
I would like to have a talking-based therapy for my sleep	13.4%	27.6%	13.4%	21.3%	24.4%	
I would like to have an activity-based therapy for my sleep	15.1%	33.3%	13.5%	15.9%	22.2%	
I would like to have a sleep treatment involving changing my light exposure	21.9%	23.4%	16.4%	20.3%	18.0%	
I would have any type of sleep therapy if it will work	27.8%	27.8%	14.3%	15.9%	14.3%	
I would like to be referred to an intervention like L-DART*	33.8%	18.1%	17.3%	10.5%	20.3%	

Service users were asked to rate or select interest in individual components of sleep interventions, and treatment related preferences and beliefs. Being prescribed a drug for sleep was least popular, and ‘I would have any type of sleep therapy if it will work’ was most endorsed. See Table 4.

Ordinal logistic regression found no demographic/diagnostic variables were associated with service users wanting to be referred. Unsurprisingly, service users with worse sleep problems were significantly more likely to want a referral (OR 1.319, p = 0.002).

Similarly, multi-level ordinal logistic regression of staff survey responses found that staff had more intention to refer service users with worse sleep problems (OR 1.660, p = < 0.000). Intention to refer was lower in NHS Trust #2 (OR 0.301, p = 0.011), if the service user had a primary diagnosis of substance misuse (OR 0.203, p = 0.019), of schizophrenia spectrum disorder (OR 0.324, p = 0.012) or diagnosis ‘none of the above’ (OR 0.116, p = 0.001).

### Qualitative comments

Free text comments on reasons not to refer / be referred were entered by a minority of respondents, usually only a few words. Comments expressed some common sentiments, including:

- medication aids sleep, so sleep therapy is unnecessary (n = 5 service users);
- sleep problems might resolve without therapy (n = 2 staff);
- therapy will not work (n = 4 service users);
- the service user does not engage enough with services (n = 5 staff);
- noting current barriers to the service user making the required changes (n = 6 staff, n = 3 service users), e.g. stimulants, bereavement, homelessness, mental state, and lifestyle / too busy to take on recommendations.

There was insufficient quantity and depth of qualitative data to merit more involved or formal analysis, and we do not consider this a mixed methods paper. Full content summary and illustrative quotes are provided in Appendix C.

## Discussion

### Summary

Staff were aware of service user’s having sleep problems, reporting these with a similarly high prevalence to service users, but gave unrealistically low reports of certain problem types. Results suggested staff were aware of insomnia type problems, but not of hypersomnia type problems, OSA, or other sleep disorders. Staff were more confident to identify sleep problems than to intervene, and confidence was particularly low among psychiatrists, and inpatient and rehabilitation staff. Reassuringly, more training regarding sleep and sleep problems was associated with higher confidence to identify and address sleep.

Overall, respondents with significant or severe sleep problems were moderately likely to want referral to an intervention like L-DART, and staff were similarly likely to want to refer. Service users’ desire for referral was most influenced by their level of sleep problems. Whilst staff readiness to refer was also influenced by factors such as profession, service, and schizophrenia spectrum diagnosis. Barriers to referral and engagement raised were reported to affect only a minority, and many were temporary or situational.

### Need for non-pharmacological sleep interventions

Some service users reported being good sleepers and some commented that medication helped with sleep. However, sleep medication was rated negatively compared to other approaches. Medication effects and timing were also endorsed as factors to address to improve sleep. Overall, this suggests medication is a good solution for some but not all cases, with potential for benefit or harm. Identifying the problem type is important to offer relevant treatment and avoid harm; hypnotics are not recommended for circadian rhythm disorders [34], and avoiding benzodiazepines is important in OSA, as they can reduce muscle tone exacerbating apnoea (although non-benzodiazepine hypnotics are now thought to be safe in OSA) [35].

Many service users wanted to improve their sleep on their own (21.6%), or with self-help advice (19.5%), and more were open to ‘help from a professional to improve my sleep’ (32.1%) than were open to ‘a therapy to help improve my sleep’ (27.9%). Service user led strategies to improve sleep, and brief interventions or advice from staff, are important. These could be developed or refined and tested, to ensure they meet the needs of mental health service users, whose sleep problems are more diverse than those of the general public. If staff are able to provide relevant advice for the problem type and context, this is invaluable for those whose problems do not require a structured therapy, or who do not wish to engage in a structured therapy.

Among the 809 service users responding or reported upon, 241 selected ‘significant sleep problems’, and 97 ‘severe…’; 339 selected ‘probably’ or ‘definitely’ wanting referral to L-DART or similar (42%). Whilst sampling biases may have inflated the percentage of service users wanting referral, even half this amount (21%) would be substantial demand. This agrees with previous qualitative studies where participants described behavioural sleep treatments as acceptable, and preferable to drug-based approaches [11, 29]. Improving sleep amongst service users of mental health trusts could bring many benefits; including better cardio-metabolic health [36], quality of life [37], and reduced relapse in psychosis [8, 38, 39], bi-polar [40] and depression [41, 42].

### Need for sleep education among staff

Staff reported limited training and confidence regarding addressing sleep, agreeing with previous research [2–4]. More training predicted higher confidence to identify and address sleep problems. However even though most reported limited training, they were despite this ‘reasonably confident’ identifying sleep problems (69.4%), and very rarely ‘unsure’ of the extent of their service user’s of sleep problems (7.1%). This may suggest staff over-confidence in assessing sleep problems. Other studies suggest staff often assess sleep informally and unsystematically [2, 4], which may explain missing awareness of OSA, night eating, or parasomnias. Prevalence studies found 12.3% [43] or 22.4% [44] of mental health outpatients had night eating syndrome, suggesting our service user self-reports were more accurate (15.3%) than staff reports (1.5%). Restless legs is similarly more prevalent (14.8%) [45] than staff reports noted (4.4%). A review of OSA prevalence research suggests OSA is present in 11-42% of patients with severe mental illness [46]. Both our service user self-report (7.4%), and especially staff report (1.4%), underestimate OSA. This supports a lack of awareness in staff, not over-reporting by service users. Improving staff’s ability to correctly identify the type of sleep problems their service users are experiencing would improve referral rates and appropriateness of referrals for all types of sleep disorders.

Facilitating health behaviour change is a complex and challenging area [21]. It is possible those staff reporting least confidence to intervene in sleep problems (psychiatrists, rehab staff, inpatient staff) may not have worse skills, but a higher awareness of skill gaps. Rehabilitation and inpatient staff may be confronted by unresolved sleep problems at night. Psychiatrists have a particular responsibility for considering differential diagnosis, which may highlight gaps in sleep knowledge, as well as being approached regarding hypnotic prescription or cessation. These groups may therefore particularly embrace sleep education.

### Barriers and facilitators to referral and engagement in an intervention like L-DART

Lifestyle and illness-related barriers were similar to those reported by staff and service users elsewhere [2, 47]. Many were potentially temporary. Attitudes such as ‘I don’t think it’s possible to improve my sleep’ may be amendable to change. More evidence of effectiveness, presented accessibly with examples, may help. Motivational interviewing appears promising for enhancing engagement and uptake in other therapies [48], and may help with updtakte of sleep therapies. Sometimes antidepressant treatment of low mood might improve motivation [49] enough to enable better utilisation of behavioural sleep treatment.

Less staff readiness to refer those using alcohol or illicit drugs is perhaps reasonable, as the intervention did not describe a means to first address substance use. Less readiness to refer service users with a schizophrenia spectrum diagnosis might be due to prescription of more sedative antipsychotics, which could be seen as already addressing sleep. However, sedating people at night without addressing circadian rhythm can lead to daytime sleepiness. Other research found staff considered sleep treatment too demanding in people with psychosis [4], and stigma is also a possible contributing factor [50]. Promoting evidence for effectiveness in this group will therefore be critical to address this potential barrier to referral, by altering beliefs about consequences of therapy. There are of course other environmental, social, and task based determinants of referral behaviour, which should be considered when designing or evaluating implementation strategies [51].

Service users endorsed “I would have any type of sleep therapy if it will work”. This underlines how far expected efficacy might predict engagement. Albeit how far participants persist must also be studied in practice. One hundred and two potentially eligible people said they would want referral if L-DART were available in clinical services, but most did not then refer to the feasibility study of L-DART (referrals n = 29, although sufficient for that study to meet its recruitment target quickly). This could be through lack of evidence for a trial intervention, distrust researchers [19], lack of time to refer, or people may just be less willing to refer than they say. Of course, it is problematic for generalisability that different people participate during trials than accept a therapy once offered in services.

### Limitations

Selection bias may have influenced the study findings. Studies of non-respondents showed differences between participants and those declining, including more drug misuse [52], and lower socio-economic status [53]. Even if response rates are high, participants may differ systematically from others, based on who the study title or promotional material appeal to [54].

This study was ostensibly about sleep so may have attracted participants interested in sleep. The service user sample may overrepresent those who were easier to engage by phone and/or less acutely unwell, although we aimed to reduce this by recruiting from inpatient wards, clozapine clinics and depot clinics. We did not record medication, which was likely a significant residual confounder in the service user sample: service users from the Trust which recruited most patients from clozapine clinic reported less severe problems. Dose and type of medication may easily affect sleep problem ratings. Similarly, we did not rate severity of ongoing psychiatric symptoms, which could have affected sleep problem scores.

Service users with poor sleep are less likely to be systematically over-represented in the staff proxy reports, since staff were instructed to report on their five most recently seen service users. Although these reports may over-represent those with higher service use, who may have worse sleep due to being more ill.

## Conclusions

Our findings suggest there is an unmet need for effective behavioural and psychological sleep interventions among mental health service users, and that staff are aware of this need, despite lacking detailed knowledge or confidence to help. Findings suggest more training would help, and may be enthusiastically received. Service user barriers to referral or engagement reported were not widespread or insurmountable. The main barrier remains the lack of availability of well evidenced sleep interventions for people with severe mental illnesses, such interventions may have great unexploited potential to improve lives, reduce morbidity and mortality, and should be a priority.

### Electronic supplementary material

Below is the link to the electronic supplementary material.

Appendix 1: Survey questions

Appendix 2: Summary demographic data from Business Intelligence at NHS Trust #1

Appendix 3: Additional Tables and Figures

## Data Availability

The datasets generated and/or analysed during the current study are not publicly available due l identifiability of individuals in small demographic and professional groups, but are available from the corresponding author on reasonable request.
