# Supplementary figures and images for: Sleep problems and referral intentions in mental health services: service user self-report and staff proxy report surveys
Source: BMC Psychiatry. 2023 Aug 10;23:583. doi: 10.1186/s12888-023-04817-6 (PMC10413589; doi:10.1186/s12888-023-04817-6)

**Appendix 1, survey questions**

**Service user survey:**


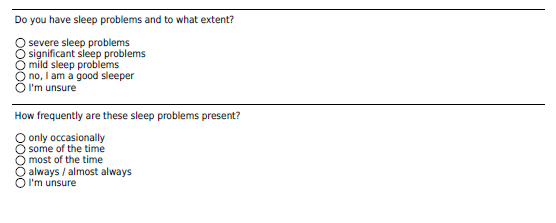


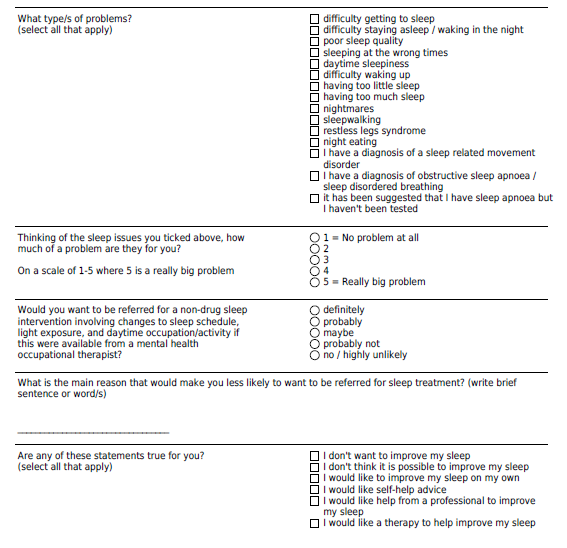


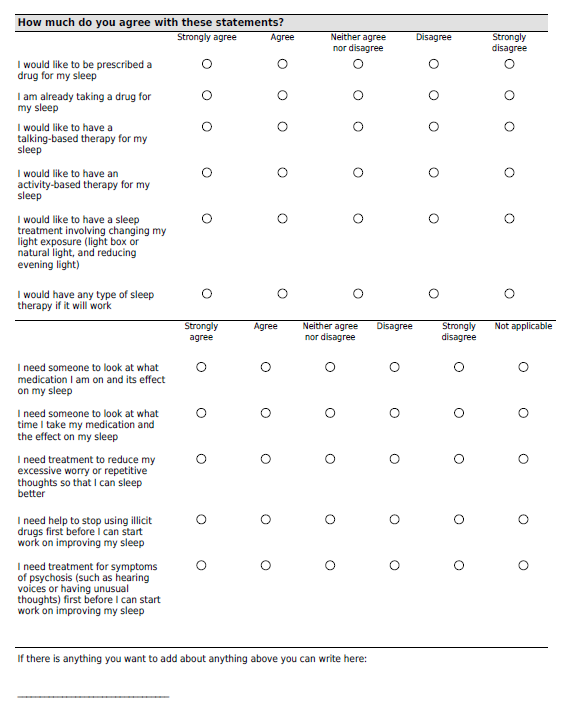

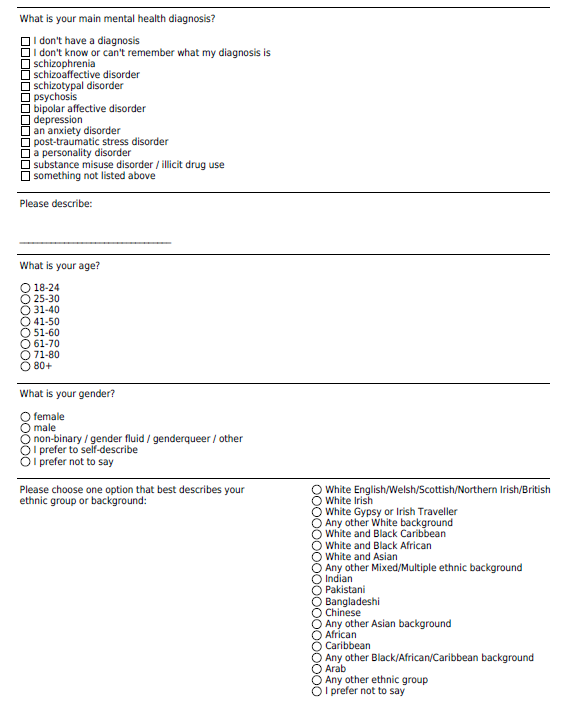

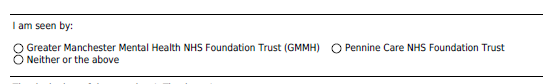


**Staff survey:**


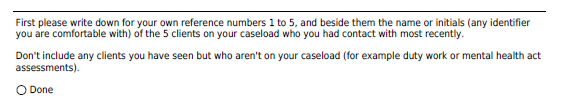


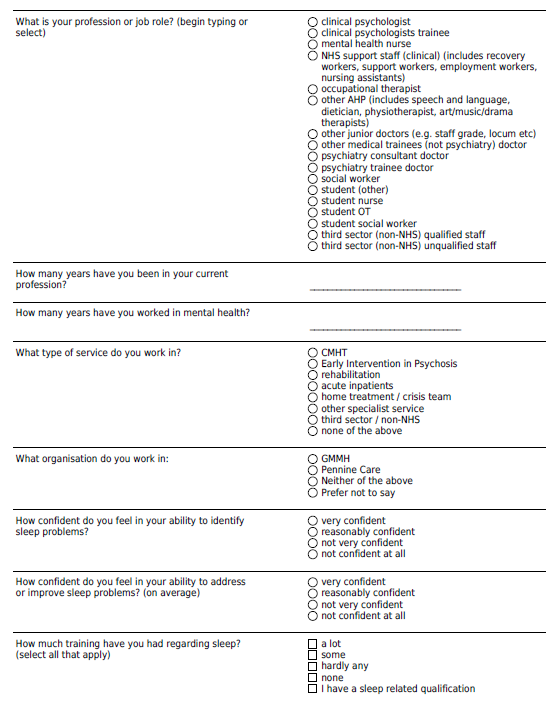


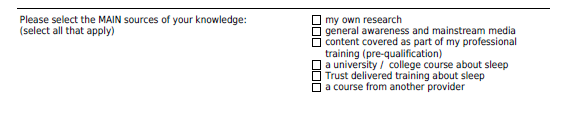

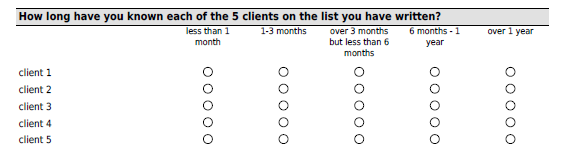


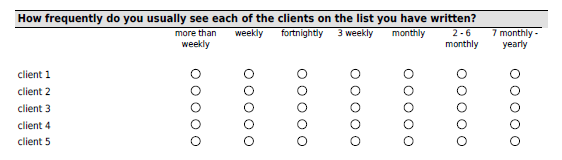


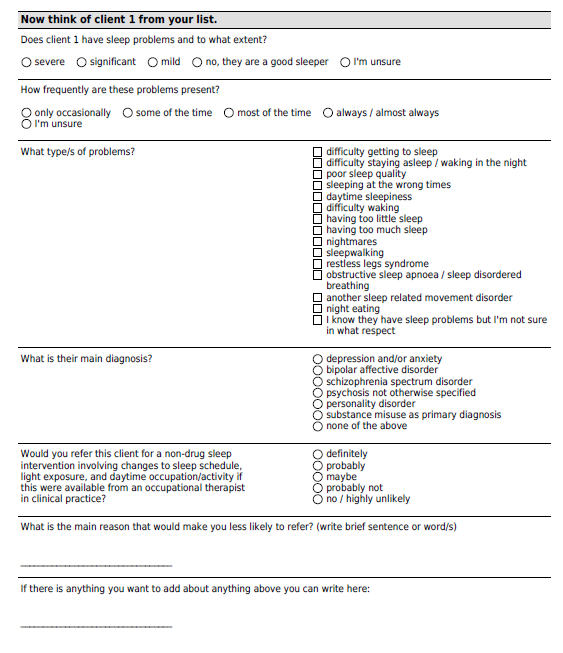

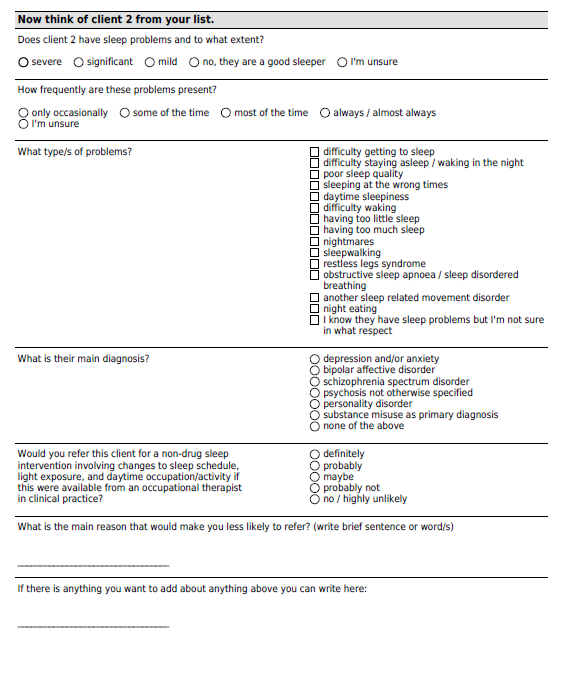


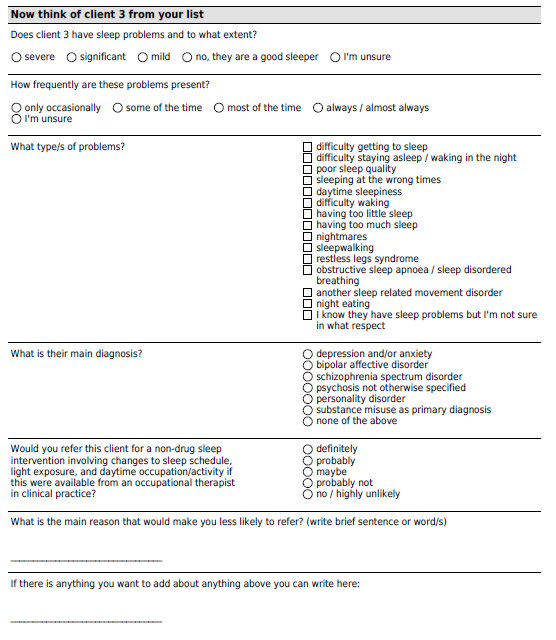


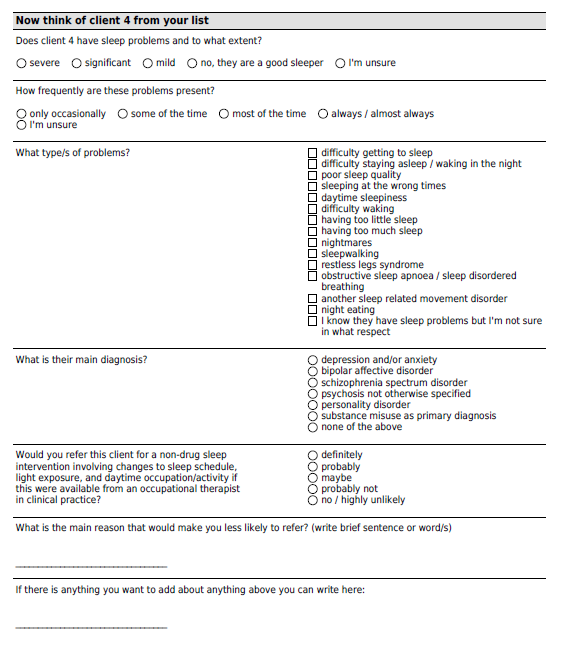


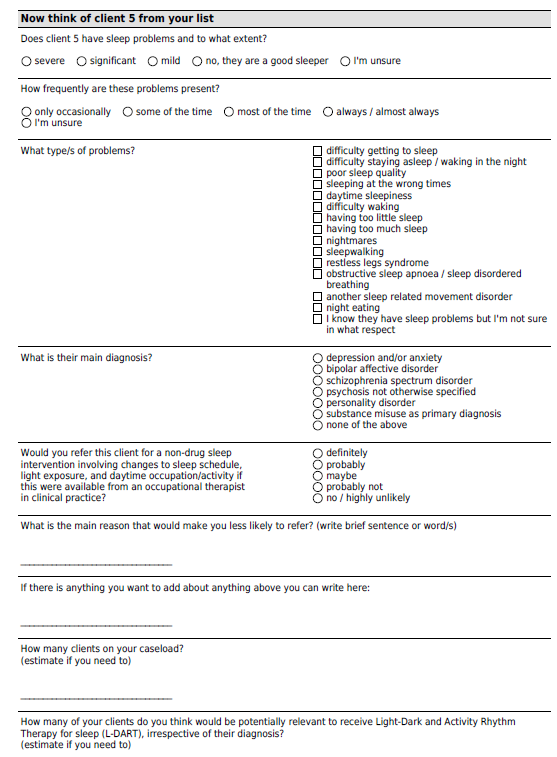


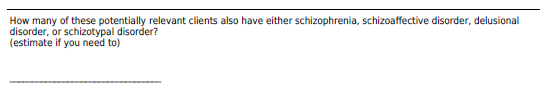

Supplement: Supplementary file 1 — Appendix 1: Survey questions [file 12888_2023_4817_MOESM1_ESM.docx]
