# Supplementary material for: Sleep problems and referral intentions in mental health services: service user self-report and staff proxy report surveys
Source: BMC Psychiatry. 2023 Aug 10;23:583. doi: 10.1186/s12888-023-04817-6 (PMC10413589; doi:10.1186/s12888-023-04817-6)
Supplement: Supplementary file 3 — Appendix 3: Additional Tables and Figures [file 12888_2023_4817_MOESM3_ESM.docx]

**Appendix 3**

[**Table C1: Service user participants diagnoses, separated by NHS Trust** 2](#_Toc88564091)

[**Table C2: Sleep problems severity service user self-report, separated by diagnosis** 2](#_Toc88564092)

[**Table C3: Sleep problems severity staff report, separated by diagnosis** 3](#_Toc88564093)

[**Table C4: Service users in whom staff are unsure if they have sleep problems, are not just those who are new to staff of infrequently seen** 3](#_Toc88564094)

[**Table C5: Staff referral intentions, separated by diagnosis** 4](#_Toc88564095)

[**Table C6: Service user wish to be referred, separated by diagnosis** 4](#_Toc88564096)

[**Table C7: Qualitative comments on reasons not to want referral** 6](#_Toc88564097)

[**Figure C1: Treatment beliefs and preferences (whole sample)** 5](#_Toc88564098)

[**Figure C2: Treatment beliefs and preferences among those who reported significant or severe sleep problems but did not want referral to an intervention like L-DART (maybe, probably not, or definitely not)** 5](#_Toc88564099)

**Table C1: Service user participants diagnoses, separated by NHS Trust**

| NHS Trust | personality disorder | PTSD | bipolar | depression / anxiety | other psychosis | schizophrenia | schizoaffective disorder | other | none | no response | Total |
| --- | --- | --- | --- | --- | --- | --- | --- | --- | --- | --- | --- |
| Trust #1 | 11  14.7% | 2  2.7% | 10  13.3% | 12  16.0% | 12  16.0% | 17  22.7% | 3  4.0% | 3  4.0% | 4  5.3% | 1  1.3% | 75 |
| Trust #2 | 2  2.0% | 0  0.0% | 4  4.0% | 7  7.1% | 4  4.0% | 66  66.7% | 7  7.1% | 1  1.0% | 3  3.0% | 5  5.1% | 99 |
| Total | 13  7.5% | 2  1.2% | 14  8.0% | 19  10.9% | 16  9.2% | 83  47.7% | 10  5.7% | 4  2.3% | 7  4.0% | 6  3.4% | 174 |

**Table C2: Sleep problems severity service user self-report, separated by diagnosis**

| **Severity of sleep problems** | Whole sample | personality disorder | PTSD | bipolar affective disorder | depression / anxiety | other psychosis diagnosis | Schizophrenia | schizoaffective disorder | Other | I don't have a diagnosis | I don't know / can't remember |
| --- | --- | --- | --- | --- | --- | --- | --- | --- | --- | --- | --- |
| no, I am  a good sleeper | 54 28.6% | 0  0.0% | 1  50.0% | 0  0.0% | 0  0.0% | 4  25.05 | 37  44.6% | 4  40% | 0  0.1% | 4  40.1% | 3  50% |
| mild  sleep problems | 54  28.6% | 1  7.7% | 0  0.0% | 2  14.3% | 6  27.3% | 4  25.0% | 29  34.9% | 3  30% | 2  40.0% | 2  20.0% | 1  16.7% |
| significant  sleep problems | 47  24.9% | 6  46.2% | 1  50.0% | 7  50.0% | 8  36.4% | 5  31.3% | 13  15.7% | 1  10.0% | 1  20.0% | 2  20.0% | 1  16.7% |
| severe  sleep problems | 32  16.9% | 5  38.5% | 0  0.0% | 5  35.7% | 8  36.4% | 3  18.8% | 4  4.8% | 2  20.0% | 1  20.0% | 2  20.0% | 1  16.7% |
| I'm unsure | 2  1.05% | 1  7.7% | 0  0.0% | 0  0.0% | 0  0.0% | 0  0.0% | 0  0.0% | 0  0.0% | 1  20.0% | 0  0.0% | 0  0% |

**Table C3: Sleep problems severity staff report, separated by diagnosis**

| **Severity of sleep problems** | Whole sample | bipolar | depression / anxiety | personality disorder | psychosis | schizophrenia | substance misuse | none of the above |
| --- | --- | --- | --- | --- | --- | --- | --- | --- |
| no, they are a good sleeper | 144  23.3% | 13  24.5% | 19  18.6% | 18  23.4% | 14  18.9% | 51  25.6% | 10  17.9% | 19  32.8% |
| mild | 172  27.8% | 14  26.4% | 42  41.2% | 23  29.9% | 27  36.5% | 41  20.6% | 19  33.9% | 6  10.3% |
| significant | 194  31.3% | 15  28.3% | 22  21.6% | 26  33.8% | 21  28.4% | 73  36.7% | 20  35.7% | 17  29.3% |
| severe | 65  10.5% | 8  15.1% | 9  8.8% | 6  7.8% | 9  12.2% | 27  13.6% | 2  3.6% | 4  6.9% |
| I'm unsure | 44  7.1% | 3  5.7% | 10  9.8% | 4  5.2% | 3  4.1% | 7  3.5% | 5  8.9% | 12  20.7% |

**Table C4: Service users in whom staff are unsure if they have sleep problems, are not just those who are new to staff of infrequently seen**

| How well known | **0** | **1** | **2** | **3** | **4** | **5** | **6** | **7** | **8** | **9** | **10** |
| --- | --- | --- | --- | --- | --- | --- | --- | --- | --- | --- | --- |
| Number of service users for whom staff selected “I'm unsure” if they have sleep problems (n) | 0 | 2 | 4 | 5 | 2 | 11 | 4 | 7 | 2 | 3 | 1 |
| % | 0.00 | 4.88 | 9.76 | 12.20 | 4.88 | 26.83 | 9.76 | 17.07 | 4.88 | 7.32 | 2.44 |
| Total of sample in this category | 8 | 9 | 16 | 40 | 52 | 102 | 115 | 94 | 69 | 52 | 30 |
| % | 1.36 | 1.53 | 2.73 | 6.81 | 8.86 | 17.38 | 19.59 | 16.01 | 11.75 | 8.86 | 5.11 |

“How well known” is a composite variable where: How long known “less than 1 month"=0, "1-3 months"=1, "over 3 months but less than 6 months"=2, "6 months - 1 year"=3, "over 1 year"=4. How often seen "7 monthly - yearly"=0, "2 - 6 monthly"=1, "monthly"=2, "3 weekly"=3, "fortnightly"=4, "weekly"=5, "more than weekly"=6

**Table C5: Staff referral intentions, separated by diagnosis**

|  | bipolar | depression | none of the above | personality disorder | psychosis not otherwise specified | schizophrenia spectrum disorder | substance misuse as primary diagnosis | Total |
| --- | --- | --- | --- | --- | --- | --- | --- | --- |
| definitely | 14  26.4% | 23  22.6% | 7  12.1% | 19  24.7% | 20  27.07% | 42  21.17% | 11  19.67% | 136  22.07% |
| probably | 10  18.9% | 20  19.6% | 4  6.9% | 16  20.8% | 20  27.0% | 47  23.6% | 17  30.4% | 134  21.7% |
| maybe | 5  9.4% | 10  9.8% | 6  10.3% | 9  11.7% | 6  8.1% | 19  9.6% | 6  10.7% | 61  9.9% |
| probably not | 6  11.3% | 13  12.8% | 5  8.6% | 7  9.1% | 7  9.5% | 13  6.5% | 6  10.7% | 57  9.2% |
| no / highly unlikely | 2  3.8% | 6  5.9% | 5  8.6% | 4  5.2% | 3  4.1% | 20  10.1% | 1  1.8% | 41  6.6% |
| Total | 53 | 102 | 58 | 77 | 74 | 199 | 56 | 619 |

**Table C6: Service user wish to be referred, separated by diagnosis**

|  | personality disorder | PTSD | bipolar affective disorder | depression / anxiety | other psychosis diagnosis | Schizophrenia | Schizoaffective disorder | I don't know / can't remember | other | none | no response | Total |
| --- | --- | --- | --- | --- | --- | --- | --- | --- | --- | --- | --- | --- |
| definitely | 5  38.5% | 1  100% | 4  28.6% | 8  38.1% | 6  50.0% | 9  19.6% | 2  7.4% | 3  100% | 1  25.0% | 2  33.3% | 2  28.6% | 44  33.1% |
| probably | 4  30.8% | 0  0.0% | 6  42.9% | 5  23.8% | 1  8.3% | 6  13.0% | 0  0.0% | 0  0.0% | 1  25.0% | 0  0.00% | 1  14.3% | 24  18.1% |
| maybe | 3  23.1% | 0  0.0% | 2  14.3% | 4  19.1% | 2  16.7% | 4  8.7% | 1  4.4% | 0  0.0% | 1  25.0% | 4  66.7% | 2  28.6% | 23  17.3% |
| probably not | 1  7.7% | 0  0.0% | 1  7.1% | 3  14.3% | 2  16.7% | 7  15.2% | 0  0.0% | 0  0.0% | 0  0.0% | 0  0.00% | 1  14.3% | 15  11.3% |
| no/ highly unlikely | 0  0.0% | 0  0.0% | 1  7.1% | 1  4.8% | 1  8.3% | 20  43.5% | 3  6.8% | 0  0.0% | 1  25.0% | 0  0.00% | 1  14.3% | 27  20.3% |
| Total | 13 | 1 | 14 | 21 | 12 | 46 | 6 | 3 | 4 | 6 | 7 | 133 |

**Figure C1: Treatment beliefs and preferences (whole sample)**

**Figure C2: Treatment beliefs and preferences among those who reported significant or severe sleep problems but did not want referral to an intervention like L-DART (maybe, probably not, or definitely not)**

**Table C7: Qualitative comments on reasons not to want referral**

| **Sentiment / content** | **example quote(s)** | **number of instances** |
| --- | --- | --- |
| Don’t need therapy to improve sleep, because sleep is not too bad | “Not too much of an issue” (staff proxy)  “I think i'm alright” (service user) | staff = 3  service users = 5 |
| Already have enough other mental health input | “Already have enough engagement with treatments of various kinds” (service user)  “Receiving treatment” (service user) | service users = 3 |
| Medication helps with sleep, don’t need a sleep therapy | “[medication] it's helping me stay asleep and not have any nightmares.” (service user)  “Only would like a short term prescription when unable to sleep.” (service user)  “clozapine helps to sleep.” (service user) | service users = 5 |
| Sleep problem expected to resolve without treatment | “As a result of medication withdrawal” (staff)  “whether they will get better […] once her psychosis is effectively treated” (staff) | staff = 2 |
| Concern that it wont work | “Probably won't work” (service user)  “Nothing can be done about my sleep.” (service user) | service users = 4 |
| Just not interested / not motivated | “lack of motivation, doesn't see it is a problem (usual for them)” (staff)  “I wouldn't be interested.” (service user) | staff = 2  service users = 1 |
| “Engagement” | “if they were willing to engage” (staff) | staff = 5 |
| Current specific barriers to making the required changes / homework | “On-going stimulant misuse” (staff)  “Wakes very early for work.” (staff)  “Recent bereavement” (staff)  “Currently NFA” (staff)  “would consider in future with more stable mood” (staff)  “I have a lot going on at the moment with my mental health. ” (service user)  “I lead a busy life and I'm not sure I would be able to engage fully in advice offered.” (service user) | staff = 6  service users = 3 |
| other concerns where there was only one instance of this content | - rather do on own (service user) - don’t like groups (assumes group therapy) (service user) - concern that if sleep is improved underlying mental health condition will not be diagnosed properly (service user) - would want more information first to decide (service user) - would refer if it was evidence based (staff) - would refer if sure service users with substance use wont be excluded (staff) | |
